# Development of Novel Methods to Define Deficits in Appendicular Lean Mass Relative to Fat Mass

**DOI:** 10.1371/journal.pone.0164385

**Published:** 2016-10-10

**Authors:** David Weber, Jin Long, Mary B. Leonard, Babette Zemel, Joshua F. Baker

**Affiliations:** 1 Division of Endocrinology and Diabetes, Golisano Children’s Hospital, University of Rochester, Rochester, United States of America; 2 Children’s Hospital of Philadelphia, Philadelphia, United States of America; 3 Department of Pediatrics and Medicine, Stanford University, Stanford, United States of America; 4 Philadelphia Veterans Affairs Medical Center, Philadelphia, United States of America; 5 University of Pennsylvania, School of Medicine, Philadelphia, United States of America; 6 Center for Clinical Epidemiology and Biostatistics, University of Pennsylvania, Philadelphia, United States of America; University of Wisconsin Madison, UNITED STATES

## Abstract

**Background:**

Recent studies suggest that adjustment of measures of lean mass for adiposity improves associations with physical function. Our objective was to develop and test a method to adjust appendicular lean mass for adiposity.

**Methods:**

Whole-body DXA data in 14,850 adults in the National Health and Nutrition Examination Survey were used to generate sex-, and race-specific standard deviation scores (Z-Scores relative to age and T-scores relative to 25 year-olds) for appendicular lean mass index (ALMI, kg/m^2^) and fat mass index (FMI, kg/m^2^). Correlations between ALMI and FMI Z- and T-Scores were assessed within demographic categories. Fat-adjusted ALMI (ALMI_FMI_) scores were determined using residual methods. Sarcopenia was defined as a T-Score <-2.0 and low lean for age as a Z-Score <-1.0. Correlations with physical function were assessed in an at-risk population.

**Results:**

Positive associations between ALMI and FMI Z- and T-Scores were significant (R >0.50; p<0.001) within all demographic categories. The impact of a unit greater FMI Z-score on ALMI Z-score was less in the elderly, men, white subjects, and among individuals with lower FMI (all tests for interaction p<0.001). There was fair agreement between ALMI and ALMI_FMI_ estimates of sarcopenia and *low lean for age* [Kappa: 0.46, 0.52, respectively (p<0.0001)]. Elderly subjects were likely to be re-classified as sarcopenic while young subjects were likely to be re-classified as normal using ALMI_FMI_. ALMI_FMI_ T-scores resulted in approximately twice the number of subjects defined as sarcopenic, compared with ALMI T-Scores. (1299 v. 534). Among rheumatoid arthritis patients, ALMI_FMI_ Z-scores correlated with physical function (Health Assessment Questionnaire: rho = -0.22, p = 0.04; Short Physical Performance Battery: rho = 0.27, p = 0.01); however, the ALMI Z-Score did not.

**Conclusions:**

Adjustment of ALMI for the confounding association with FMI impacts the definition of lean mass deficits. These methods provide a practical tool for investigators and clinicians based on population-based reference data.

## Introduction

Sarcopenia, or low muscle mass, as a consequence of aging or chronic disease, is associated with impaired physical function [1]. Early studies defined sarcopenia as a low appendicular lean mass index (ALMI, kg/ht^2^) as measured by DXA, compared with a young reference population [2]. Numerous subsequent studies demonstrated substantial variability in the prevalence of sarcopenia and correlations with clinical outcomes when based on different definitions [3].

Greater fat mass is also associated with impaired physical function [4]. Complicating matters, individuals with greater fat mass typically have greater skeletal muscle mass [5]. A greater ALMI in association with greater fat mass index (FMI) does not translate into proportional gains in strength, suggesting lesser muscle quality [6]. Thus adiposity is an important confounder that may mask true relationships between physical functioning and ALMI. In other words, the lack of consideration of adiposity in previous definitions of sarcopenia resulted in a potential underestimation of the impact on physical functioning.

Several previous studies demonstrated that estimates of muscle mass adjusted for fat mass showed stronger associations with lower extremity function and better prediction of incident mobility limitations, compared with methods that failed to consider the confounding effects of adiposity [7–9]. The Foundation for National Institutes of Health (FNIH) Sarcopenia Project recently developed diagnostic criteria for sarcopenia [10]. Sensitivity analyses indicated that obesity influenced the relation between appendicular lean mass (ALM, kg) and muscle strength; therefore, alternate cut-points were derived using an ALM-to-BMI ratio (m^2^). This measure was associated with mobility impairment while ALM alone was not [11]. Further refinement of this approach should consider sex and race/ethnic differences and non-linear associations between ALM and fat mass that are not captured by the ALM-to-BMI ratio or other published methods. To date, studies in this area have not provided the data necessary for investigators to apply these adjustments, and none used a nationally representative reference population.

The majority of existing definitions of sarcopenia are based on a single cut-point for ALM or ALMI, typically based on the values in healthy young adults. This is analogous to the use of a T-score cutoff for bone mineral density to define osteoporosis. However, the origins of muscle deficits are complex and may occur as a result of aging, chronic disease, or both. In order to investigate these effects, it is essential to facilitate comparisons to both age-specific (i.e. Z-score) and healthy young adult (i.e. T-score) populations. These measures can then be used to develop more comprehensive definitions of sarcopenia that capture variations with age, sex, race/ethnicity and fat mass (and their interactions) to capture both the impact of aging and chronic disease.

This study aims to accomplish the above by leveraging DXA body composition data available in a diverse sample of 14,850 participants (7,333 female) aged 20 to 85 years through the National Health and Nutrition Examination Survey (NHANES). Specifically, the objectives were 1) to characterize associations between ALMI and FMI within age, sex, and race/ethnic groups, 2) to establish new sex- and race- specific definitions for low ALMI adjusted for the confounding effects of FMI (ALMI_FMI_) compared to normal young adults (T-Scores) and within age groups (Z- Scores), 3) to determine how adjustment for FMI impacts the classification of individuals as having low ALMI and sarcopenia, and 4) to illustrate the utility of fat-adjusted measures of ALMI in an at risk group. Our overall goal was to provide investigators and clinicians with practical tools to generate sex and race-specific T and Z-scores for ALMI with adjustment for FMI.

## Methods

### Development of Fat-Adjusted Measures of Appendicular Lean Mass Index

This study utilized whole-body DXA data from 1999–2006 NHANES in adults ≥ 20 years of age [12]. NHANES is an annual survey conducted by the National Center for Health Statistics (NCHS) that uses a complex, multistage probability sampling method including oversampling of non-Hispanic blacks, Mexican Americans, and low-income whites. The survey included a household interview and a detailed examination in mobile examination centers. Approval for NHANES 1999–2006 was obtained from the NCHS Institutional Review Board (IRB); a waiver of IRB oversight was obtained from the Research Subjects Review Board at the University of Rochester.

Whole-body DXA scans were obtained using a Hologic QDR 4500A fan beam densitometer (Hologic, Inc. Bedford, MA) in eligible participants. All DXA scans were reviewed and analyzed by the University of California, San Francisco, Radiology department using Hologic Discovery Software, version 12.1. Exclusion criteria included pregnancy, weight >300 pounds (136 kg), height >77 inches (195 cm), recent nuclear medicine scan or exposure to radioactive contrast. Multiple imputation of missing data was performed by the NCHS to address the potential biases of nonrandom missing DXA data. Full details of the methods and rationale for multiple imputation are described elsewhere [13].

Age was calculated in months as reported at the time of examination. US Census Bureau classifications for race and ethnicity were ascertained by participant self-report. Height (cm) was obtained by using standard procedures using a fixed stadiometer. FMI and ALMI (kg/m^2^) were calculated from DXA-measured body-composition data, excluding bone mineral content. A prior multicenter analysis of DXA body composition data showed an overestimation of lean body mass and underestimation of fat mass by Hologic QDR 4500A fan-beam densitometers [14]. Accordingly, NHANES DXA body-composition data for fat mass and lean body mass were adjusted by the NCHS such that lean body mass was decreased by 5% and fat mass increased by an equivalent amount (kg) to maintain total body mass.

### Sub-Study of Correlation of Body Composition Measures with Physical Function

Fat-adjusted ALMI Z-Scores were evaluated in a cohort of 111 subjects with rheumatoid arthritis (RA) from the University of Pennsylvania and the Philadelphia Veterans Affairs Medical Center. Details of this study population and study methods have been described.[15, 16] This RA cohort was developed to evaluate alterations in body composition and bone structure in patients with RA. Subjects consisted of individuals with RA, ages 18–70 years, who met 2010 American College of Rheumatology criteria. Whole-body DXA scans were obtained using an Hologic densitometer (Delphi Systems, *Hologic*, *Inc*., Bedford, MA). The NHANES Body Composition Assessment (BCA) calibration was applied to adjust for systematic over- and under- estimation of body composition measures using DXA [14], as described above. Disability was measured using the Health Assessment Questionnaire [17], a well-validated measure of disability utilized for clinical trials in RA. The score ranges from 0–3 with higher scores representing greater disability and functional impairment. Physical functioning was also assessed using the Short Physical Performance Battery (SPPB). The SPPB is a widely used and simple test to measure lower extremity function through observed completion of tasks that mimic daily actions. Specifically it examines an individual’s performance with regard to static balance, gait speed, and timed chair-raises [18]; each component is scored between 0–4 for a total of 12 possible points with higher scores indicating better physical function.

### Statistical Analysis

Statistical analysis was performed using Stata 13.0 (*StataCorp*, *LP*, *College Station*, *TX*) and SAS version 9.3 (*SAS Institute*, *Cary*, *NC*). Correlations between measures of ALMI and FMI were assessed using Pearson’s correlations and linear regression models within each decade of age, stratified by sex and race. Testing for differing associations within subgroups (i.e. age, sex, race) was performed by evaluating the significance of multiplicative interaction terms. Analyses included imputed data and were performed using sample weights to account for the complex sample design as recommended by the NCHS [19].

#### Adjustment of ALMI for Total FMI

The *lambda-mu-sigma* (LMS) method is the gold standard for generating standard deviation scores in datasets characterized by non-linearity, heteroskedasticity, and skew, such as ALMI and FMI relative to age[20]. Conventional sex- and race/ethnicity-specific Z-Scores were generated for FMI relative to age using LMS curves previously reported by *Hologic Inc*.[21, 22] and for ALMI relative to age using LMS curves provided by *Hologic* (personal communication). In addition, T-scores were generated for each individual using the LMS values in the 25 year-old participants. Of note, sex-specific ALMI and FMI results compared with young adult and age-matched NHANES participates are now routinely provided on DXA clinical reports.

We developed a novel method to generate fat-adjusted ALMI (ALMI_FMI_**)** Z-Scores by obtaining residuals from the regression of ALMI Z-Score on FMI Z-Score within age, sex, and race categories. Similarly, ALMI_FMI_ T-Scores were determined by obtaining residuals from the regression of ALMI T-Score on FMI T-Score among 20–40 year-olds. The relations between ALMI and FMI Z-scores, and ALMI and FMI T-Scores were non-linear and inclusion of the significant FMI^2^ term improved the model fit. Failure to adjust for this relation resulted in an over-estimate of residuals at the extremes of adiposity, as illustrated below. The resultant ALMI_FMI_ Z- and T-Scores were standardized to achieve an SD of 1.0 within all groups. Correlations between ALMI and ALMI_FMI_ Z-Scores and T-Scores were determined using Pearson’s correlations.

#### Definitions of “Low Lean Mass for Age” and “Sarcopenia”

In unadjusted analyses, “*low lean for age*” was defined as a sex and race/ethnicity specific ALMI Z-Score of ≤ -1 (equivalent to the 16th percentile for age). “Sarcopenia” was defined as a sex and race/ethnicity specific T-score ≤ -2 (equivalent to the 2^nd^ percentile in 25 year old), consistent with prior definitions [2, 23, 24]. For the fat-adjusted analyses “low fat-adjusted lean for age” was defined as ALMI_FMI_ Z-score ≤ -1. “Fat-adjusted sarcopenia” was defined as ALMI_FMI_ T-score ≤ -2. Agreement between standard and fat-adjusted definitions of “low lean mass for age” and “sarcopenia” was assessed using kappa statistics.

#### Comparison of Standard and Fat-Adjusted Lean Measures with Functional Outcomes

In order to assess the construct validity of the fat-adjusted measures, relationships were assessed between ALMI and ALMI_FMI_ standard deviation scores and the results of the HAQ and SPPB. Because SPPB scores are highly skewed, this variable was analyzed as categories as previously defined (0–4, 5–8, and 9–12) [25, 26]. Linear and ordinal regression models assessed associations independent of age and sex.

## Results

### Assessment of Associations between ALMI and FMI

The characteristics of the NHANES and RA study cohorts are summarized in **Table A in S1 File**. Associations between ALMI Z-Scores (per 1 SD) and FMI Z-Scores (per 1 SD) within NHANES participants are shown in **Table 1**. ALMI and FMI Z-Scores were strongly and positively correlated within all age, sex and race strata (all p<0.001) and significant FMI^2^ terms suggested weaker associations at lower FMI Z-Scores. The ALMI-FMI Z-score relations differed with age (significant age-FMI Z-score interaction, p <0.01): a unit greater FMI Z-Score was associated with a lesser increment in ALMI Z-Score in older participants. In addition, there was a significant interaction with sex and race such that associations were less pronounced among men and white participants (p for interaction <0.001). For example, among 60–70 year-old white women, the beta-coefficient for FMI Z-Score was 0.77 while, in contrast, among 60–70 year-old white men, it was only 0.64.

**Table 1:** Regressions of FMI on ALMI Z-Score within age, sex, and race categories.

**MALES**	**20–30 (n = 1311)**		**30–40 (n = 1293)**		**40–50 (n = 1436)**		**50–60 (n = 1115)**		**60–70 (n = 1264)**		**70–90 (n = 1098)**	
	**β (95%CI)**	**p**	**β (95%CI)**	**p**	**β (95%CI)**	**p**	**β (95%CI)**	**p**	**β (95%CI)**	**p**	**β (95%CI)**	**p**
**White**												
**FMIZ**	0.68 (0.62,0.75)	<0.001	0.61 (0.54,0.67)	<0.001	0.64 (0.57,0.72)	<0.001	0.65 (0.57,0.74)	<0.001	0.64 (0.57,0.7)	<0.001	0.51 (0.45,0.56)	<0.001
FMIZ*FMIZ	0.04 (-0.01,0.08)	0.06	0.12 (0.07,0.16)	<0.001	0.1 (0.06,0.15)	<0.001	0.03 (-0.01,0.08)	0.18	0.05 (-0.02,0.11)	0.14	0.02 (-0.02,0.06)	0.35
**Constant**	-0.09 (-0.15,-0.02)	0.01	-0.1 (-0.17,-0.03)	0.004	-0.06 (-0.15, 0.02)	0.13	-0.05 (-0.14,0.04)	0.26	-0.02 (-0.12,0.07))	0.65	-0.04 (-0.12,0.04)	0.31
**SD**	0.72		0.76		0.74		0.76		0.81		0.82	
**Black**												
**FMIZ**	0.64 (0.58,0.71)	<0.001	0.69 (0.61,0.77)	<0.001	0.75 (0.66,0.85)	<0.001	0.75 (0.65,0.84)	<0.001	0.69 (0.6,0.79)	<0.001	0.69 (0.55,0.84)	<0.001
FMIZ*FMIZ	0.13 (0.06,0.2)	<0.001	0.15 (0.09,0.21)	<0.001	0.11 (0.04,0.17)	0.002	0.1 (0.03,0.17)	0.01	-0.003 (-0.07,0.06)	0.94	-0.01 (-0.09,0.08)	0.87
**Constant**	-0.20 (-0.32,-0.07)	0.003	-0.11 (-0.21,-0.01)	0.03	-0.1 (-0.19,-0.01)	0.03	-0.08 (-0.19,0.03)	0.17	0.05 (-0.06,0.15)	0.37	-0.08 (-0.23,0.08)	0.35
**SD**	0.67		0.69		0.66		0.69		0.72		0.68	
**MexAm**												
**FMIZ**	0.64 (0.56,0.73)	<0.001	0.72 (0.61,0.83)	<0.001	0.68 (0.59,0.78)	<0.001	0.68 (0.58,0.78)	<0.001	0.61 (0.53,0.70)	<0.001	0.51 (0.39,0.62)	<0.001
FMIZ*FMIZ	0.09 (0.04,0.14)	<0.001	0.04 (-0.01,0.08)	0.07	0.04 (-0.02,0.11)	0.16	0.06 (-0.02,0.13)	0.13	0.08 (0.03,0.13)	0.001	0.09 (0.01,0.18)	0.06
**Constant**	-0.13 (-0.24,-0.03)	0.01	-0.03 (-0.14,0.08)	0.56	-0.09 (-0.18,-0.01)	0.03	-0.1 (-0.24,0.03)	0.14	-0.06 (-0.17,0.04)	0.24	-0.17 (-0.33,-0.02)	0.03
**SD**	0.73		0.77		0.80		0.74		0.80		0.78	
**FEMALES**	**20–30 (n = 1195)**		**30–40 (n = 1239)**		**40–50 (n = 1435)**		**50–60 (n = 1093)**		**60–70 (n = 1305)**		**70–90 (n = 1066)**	
	**β (95%CI)**	**p**	**β (95%CI)**	**p**	**β (95%CI)**	**p**	**β (95%CI)**	**P**	**β (95%CI)**	**p**	**β (95%CI)**	**p**
**White**												
**FMIZ**	0.84 (0.77,0.91)	<0.001	0.73 (0.67,0.78)	<0.001	0.75 (0.69,0.8)	<0.001	0.72 (0.67,0.78)	<0.001	0.77 (0.72,0.82))	<0.001	0.7 (0.65,0.76)	<0.001
FMIZ*FMIZ	0.12 (0.04,0.19)	0.003	0.18 (0.12,0.23)	<0.001	0.14 (0.10,0.19)	<0.001	0.14 (0.11,0.17)	<0.001	0.08 (0.05,0.11)	<0.001	0.09 (0.05,0.13)	<0.001
**Constant**	-0.13 (-0.23,-0.03)	0.01	-0.18 (-0.26,-0.09)	<0.001	-0.11 (-0.19,-0.03)	0.008	-0.16 (-0.24,-0.09)	<0.001	-0.1 (-0.18,-0.03)	0.006	-0.1 (-0.17,-0.02)	0.01
**SD**	0.65		0.64		0.66		0.64		0.63		0.71	
**Blacks**												
**FMIZ**	0.86 (0.79,0.93)	<0.001	0.82 (0.74,0.91)	<0.001	0.81 (0.74,0.88)	<0.001	0.88 (0.80,0.96)	<0.001	0.78 (0.70,0.87)	<0.001	0.69 (0.60,0.78)	<0.001
FMIZ*FMIZ	0.11 (0.04,0.18)	0.002	0.1 (0.04,0.15)	0.001	0.04 (0.01,0.07)	0.016	0.06 (-0.01,0.12)	0.09	0.02 (-0.04,0.08)	0.45	0.07 (0.01,0.13)	0.03
**Constant**	-0.2 (-0.33,-0.07)	0.002	-0.11 (-0.21,-0.01)	0.03	0.01 (-0.07,0.09)	0.80	-0.20 (-0.31,-0.09)	<0.001	0.02 (-0.09,0.13)	0.70	-0.07 (-0.21,0.07)	0.35
**SD**	0.58		0.59		0.56		0.66		0.63		0.64	
**MexAM**												
**FMIZ**	0.77 (0.7,0.84)	<0.001	0.74 (0.63,0.84)	<0.001	0.72 (0.64,0.80)	<0.001	0.78 (0.69,0.87)	<0.001	0.72 (0.60,0.84)	<0.001	0.63 (0.55,0.71)	<0.001
FMIZ*FMIZ	0.09 (0.03,0.15)	0.006	0.08 (-0.01,0.15)	0.05	0.08 (0.01,0.14)	0.02	0.07 (0.02,0.11)	0.003	0.11 (0.05,0.18)	0.001	0.04 (-0.02,0.09)	0.15
**Constant**	-0.16 (-0.29,-0.04)	0.01	-0.11 (-0.25,0.02)	0.09	0.02 (-0.08,0.12)	0.70	-0.09 (-0.21,0.02)	0.10	-0.12 (-0.24,-0.01)	0.04	0.01 (-0.17,0.19)	0.91
**SD**	0.67		0.70		0.63		0.70		0.62		0.65	

Abbreviations: CI = Confidence Interval; FMIT = Fat Mass Index T-Score; SD = Standard Deviation of adjusted score within the strata.

The steps required to generate an ALMI_FMI_ Z-score for an individual patient/participant using this method are detailed below.

1. Convert the ALMI and FMI results to standard deviation scores. Means and standard deviations for ALMI and FMI within age, sex, and race groups in NHANES are provided in **Tables B-E in S1 File** allow for the generation of ALMI and FMI Z-and T-Scores for individuals.
2. Determine predicted ALMI Z-Score relative to FMI Z-score using the age, sex, and race appropriate regression analyses provided in Table 1:
  $$Predicted\;ALMI\;Z\;Score=\beta_{1}(FMI\;Z\;Score)+{\;\beta }_{2}{\left(FMI\;Z\;Score\right)}^{2}+constant$$
3. Generate an ALMI_FMI_ Z-Score for the individual incorporating the actual ALMI Z-score from step 1 above, and the sex, and race appropriate SD from Table 1 using the following equation:

$$ALMI\;Z\;Score=(Actual\;ALMI\;Z\;Score-Predicted\;ALMI\;Z\;Score*\;(\frac{1}{SD})$$

For example, 65 year-old white female with an ALMI Z-Score of 0.00 and a FMI Z-Score of +1.00 would have an ALMI_FMI_ Z-Score of -1.19, or an actual value 1.19 SD below the predicted value for a subject of that age, sex, race, and FMI Z-Score.

$$Predicted\;ALMI\;Z\;Score=0.77(1.00)+0.08{\left(1.00\right)}^{2}-0.10=0.75$$

$${ALMI}_{FMI}Z\;Score=(0.00-0.75)\;\left(\frac{1}{0.63}\right)=-1.19$$

A 45 year-old black male with an ALMI Z-Score of -2.00 and a FMI Z-Score of -2.00 would have an ALMI_FMI_ Z-Score of -1.27. Of note, if were one to incorrectly assume the association with FMI Z-Score were linear (i.e. using a regression equation that was derived without the FMI^2^ term [equation not shown]), the adjusted Z-Score would be overestimated at -0.76.

$$Predicted\;ALMI\;Z\;Score=0.75(-2.00)+0.11({-2.00)}^{2}-0.10=1.16$$

$${ALMI}_{FMI}\;Z\;Score=(-2.00-(-1.16))\;\left(\frac{1}{0.66}\right)=-1.27$$

Associations between ALMI T-Scores and FMI T-Scores among adults, ages 20–40 years old, stratified by sex and race, are provided in **Table 2**. As with the above analyses, there was a significant interaction with sex and race such that associations were stronger among women (p for interaction <0.001) and black participants (p for interaction = 0.02). The same 65 year-old white female from the previous example would have an ALMI T-Score of -0.16 and a FMI T-Score of 1.35 based on NHANES reference ranges. Based on the observations in **Table 2**, she would have an adjusted ALMI T-Score of -2.00, or an actual value 2 SD below the predicted value for a 25 year-old of the same sex, race, and adiposity.

$$Predicted\;ALMI\;T\;Score=0.78(1.35)+0.18{\left(1.35\right)}^{2}-0.22=1.16$$

$${ALMI}_{FMI}T\;Score=(-0.16-1.16)\;\left(\frac{1}{0.66}\right)=-2.00$$

**Table 2:** Regression of FMI on ALMI Z-Scores within 20–40 year-olds.

**MALES**	**20–40 year olds**	
	**β (95%CI)**	**p**
**White (n = 1261)**		
**FMIT**	0.64 (0.59,0.68)	<0.001
FMIT*FMIT	0.08 (0.05,0.11)	<0.001
**Constant**	-0.14 (-0.19,-0.09)	<0.001
**SD**	0.73	
**Black (n = 637)**		
**FMIT**	0.64 (0.59,0.69)	<0.001
FMIT*FMIT	0.15 (0.1,0.19)	<0.001
**Constant**	-0.2 (-0.28,-0.12)	<0.001
**SD**	0.66	
**MexAm (n = 782)**		
**FMIT**	0.67 (0.61,0.73)	<0.001
FMIT*FMIT	0.08 (0.05,0.11)	<0.001
**Constant**	-0.1 (-0.18,-0.03)	0.009
**SD**	0.76	
**FEMALES**	**20–40 year olds**	
	**β (95%CI)**	**p**
**White (n = 1196)**		
**FMIT**	0.78 (0.73,0.83)	<0.001
FMIT*FMIT	0.18 (0.14,0.23)	<0.001
**Constant**	-0.22 (-0.29,-0.16)	<0.001
**SD**	0.66	
**Blacks (n = 641)**		
**FMIT**	0.84 (0.79,0.89)	<0.001
FMIT*FMIT	0.12 (0.08,0.16)	<0.001
**Constant**	-0.25 (-0.34,-0.16)	<0.001
**SD**	0.59	
**MexAm (n = 667)**		
**FMIT**	0.75 (0.69,0.82)	<0.001
FMIT*FMIT	0.09 (0.03,0.14)	0.001
**Constant**	-0.16 (-0.25,-0.07)	0.001
**SD**	0.7	

Abbreviations: CI = Confidence Interval; FMIT = Fat Mass Index T-Score; SD = Standard Deviation

### Agreement Between Standard and Adjusted Measures

**Fig 1:**
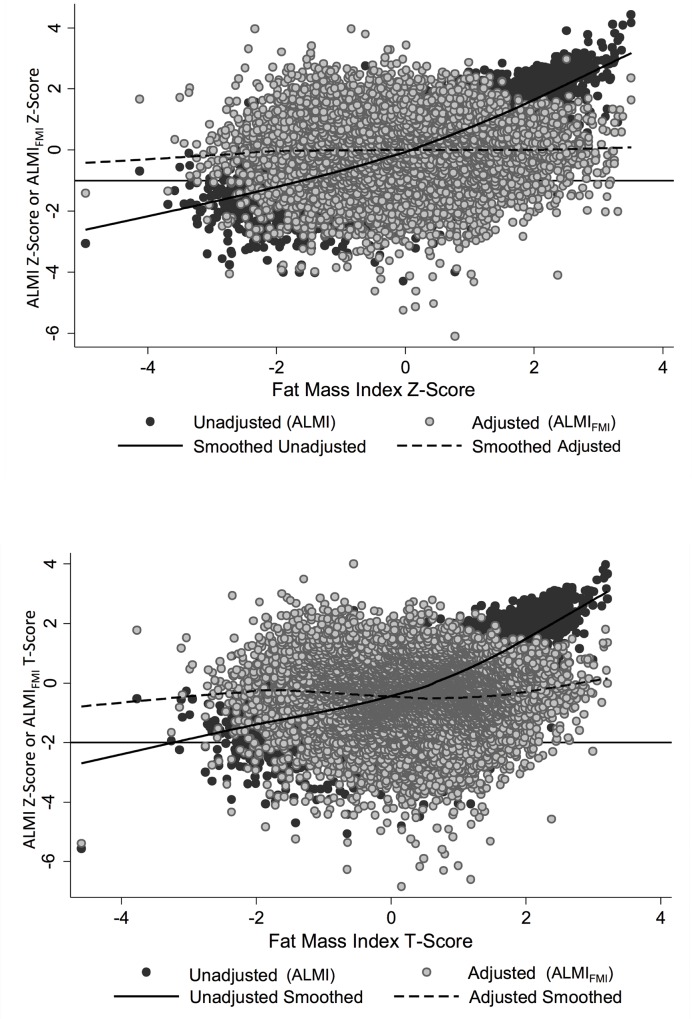
ALMI Z-Scores and T-Scores are strongly positively correlated with FMI Z-Scores and T-Scores. The scatter plots and lowess curves illustreate a) the correlation between lean/adjusted lean and fat Z -score, with cutoff of -1 displayed as line. b) the correlation between lean/adjusted lean and fat-T-score with cutoff of -2 as line.

The correlation between ALMI and ALMI_FMI_ Z-Scores was moderate [R = 0.68, p<0.001]. Correlation between ALMI and ALMI_FMI_ T-Scores was also moderate [R = 0.72, p<0.001]. **Fig 1A and 1B** demonstrate the distribution of both ALMI and ALMI_FMI_ Z-Scores and T-Scores over the range of adiposity. While the unadjusted ALMI Z-Scores and T-Scores correlate strongly with FMI Z- and T-Scores, the adjusted scores, as expected, demonstrated no correlation with FMI Z- and T-Scores.

The agreement between definitions of *low lean for age* (Z-Score <-1) was “good” between the standard ALMI and ALMI_FMI_ Z-Scores (Kappa = 0.54) (**Table 3**). As expected, 2288 (15.4%) subjects were *low lean for age* based on the standard definition. Also as would be expected, a similar number [2133 (14.4%)] were defined as *low lean for age* after fat-adjustment. However, these are not the same individuals. With fat-adjustment, a substantial proportion of the total population (1731/14,850, 11.7%) would be reclassified. Among those who would be defined as *low lean for age* based on the standard ALMI Z-Score, 41.2% (943/2288) would be re-classified as normal based on the ALMI_FMI_ Z-Score. In addition, 6.3% (788/12,562) of subjects classified as normal based on the standard ALMI Z-Score would be re-classified as *low lean for age* based on the ALMI_FMI_ Z-Score.

**Table 3:** Assessment of the correlation between standard and fat-adjusted measures of low lean mass for age (as defined as an ALMI greater than 1 SD below the mean).

	Low Lean for Age	Normal Lean for Age	Total
	(ALMI Z ≤ -1.0 SD)	(ALMI Z > -1.0 SD)	
**Low Fat-Adjusted ALMI for Age** • **(ALMI_FMI_ Z ≤ -1.0 SD)**	1345 (9.1%)	788 (5.3%)	2133 (14.4%)
**Normal Fat-Adjusted ALMI for Age** • **(ALMI_FMI_ Z > -1.0 SD)**	943 (6.4%)	11774 (79.3%)	12717 (85.6%)
Total	2288 (15.4%)	12562 (84.6%)	14850 (100%)
	Kappa = 0.54*		

*P<0.0001

Abbreviations: ALMI = Appendicular Lean Mass Index; SD = Standard Deviation; ALMI_FMI_ = Appendicular Lean Mass Index adjusted for Fat Mass Index

Agreement between the definitions of sarcopenia (T-Score <-2) are demonstrated in **Table 4**. Agreement between standard and fat-adjusted definitions was “good” (Kappa = 0.46). Use of ALMI_FMI_ T-scores resulted in approximately twice the number of subjects defined as sarcopenic overall, compared with ALMI T-scores (1299 v. 534) (**Table 4**). 16.9% (90/534) of subjects who were defined as sarcopenic based on the standard ALMI T-Score would be re-classified to normal based on the ALMI_FMI_ T-Score. In addition, 6% (855/14,316) of subjects previously classified as normal based on the standard ALMI T-Score would be re-classified as sarcopenic based on the ALMI_FMI_ T-Score.

**Table 4:** Assessment of the concordance between standard and fat-adjusted measures of sarcopenia (as defined as an ALMI greater than 2 SD below the mean for 20–40 year-olds).

	Sarcopenia	Normal ALMI T	Total
	(ALMI T ≤ -2.0 SD)	(ALMI T > -2.0 SD)	
**Fat-Adjusted Sarcopenia** • **(ALMI_FMI_ T ≤-2.0 SD)**	444 (3.0%)	855 (5.7%)	1299 (8.8%)
**Normal Fat-Adjusted ALMI T** • **(ALMI_FMI_ T >-2.0 SD)**	90 (0.6%)	13461 (90.7%)	13551 (91.3%)
Total	534 (3.6%)	14316 (96.4%)	14850 (100%)
	Kappa = 0.46*		

*P<0.0001

Abbreviations: ALMI = Appendicular Lean Mass Index; SD = Standard Deviation; ALMI_FMI_ = Appendicular Lean Mass Index adjusted for Fat Mass Index

**Fig 2:**
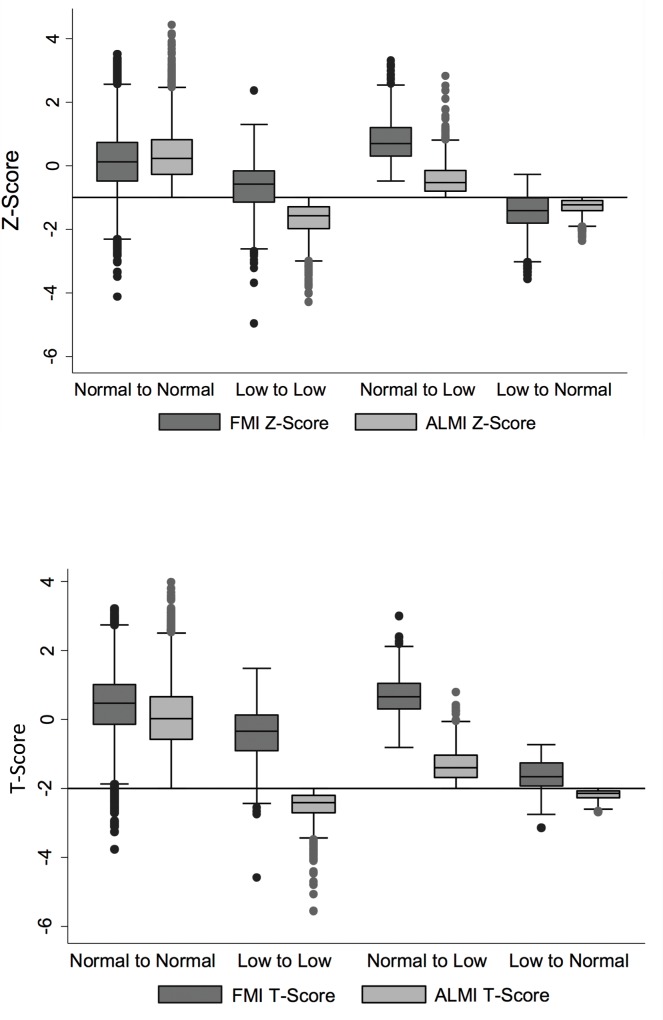
Reclassification of individuals from normal lean mass to low lean mass occurs among those with high FMI. Fig 2a demonstrates a box plot of FMI (dark grey) and ALMI (light grey) Z-Scores among subjects who were reclassified with the fat-adjusted definitions of low lean. Fig 2b is a box plot of FMI (dark grey) and ALMI (light grey) T-Scores among subjects who were reclassified with the fat-adjusted definitions of sarcopenia.

**Fig 3:**
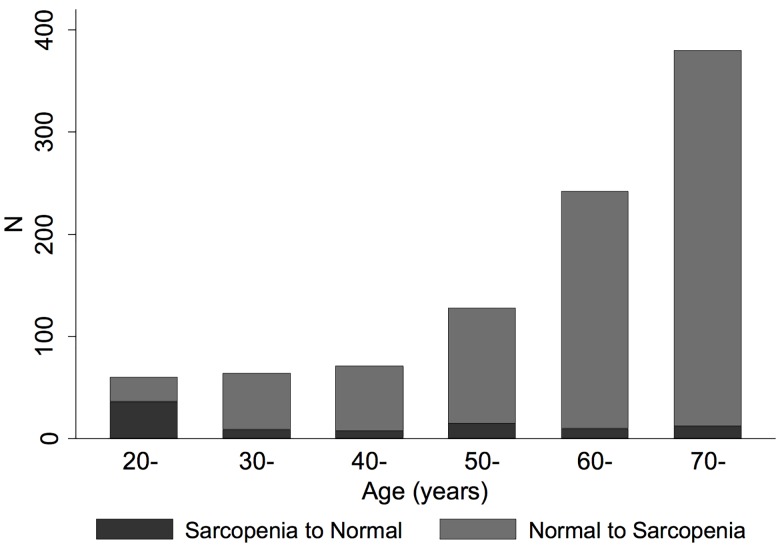
Younger individuals are likely to be reclassified as normal based on ALMI_FMI_, while elderly individuals are likely to be reclassified as having low lean mass. Illustration of subjects that were reclassified with the fat-adjusted definition of sarcopenia across the age range demonstrating greater re-classification to sarcopenia among older individuals.

**Fig 2A** demonstrates high FMI Z-Scores among those who were re-classified from normal using ALMI Z-score to *low lean for age* using ALMI_FMI_ Z-score [median 0.70 (IQR: 0.29, 1.19)]. Similarly, Fig 2B demonstrates that FMI T-Scores were high among those who were re-classified from normal using ALMI T-score to sarcopenia using ALMI_FMI_ T-score [median 0.66 (IQR: 0.3, 1.04)]. Conversely, FMI Z-Scores were low among those who were reclassified from *low lean for age* to normal [Median -1.41 (-1.82, -1.01)] and FMI T-Scores were low among those who were reclassified from sarcopenic to normal [median -1.67 (IQR: -1.94, -1.26)]. **Fig 3** illustrates that reclassification to sarcopenia with ALMI_FMI_ T-Scores occurred primarily among those with greater age. In contrast, the proportion switching from sarcopenia to normal was greater among younger subjects.

### Correlation of Standard and Fat-Adjusted Measures with Physical Function

Among 111 patients with RA, the mean (SD) HAQ score was 0.79 (0.63), and the median (IQR) for the SPPB was 11 (9, 12). The ALMI Z-Score was not associated with HAQ (R: -0.018, p = 0.85). In contrast, the ALMI_FMI_ Z-Score was inversely associated with HAQ [R: -0.29 (p = 0.002)] (p for comparison <0.001). ALMI_FMI_ Z-scores were associated with age, sex in RA participants. The association between ALMI_FMI_ and HAQ was similar in models adjusting for these covariates [β: -0.17 (-0.28, -0.059) p = 0.003].

The ALMI Z-Score was not associated with SPPB (rho: -0.054, p = 0.61), while the ALMI_FMI_ was significantly correlated with SPPB (rho: 0.27, p = 0.01). In ordinal regression models adjusted for age and sex, greater ALMI_FMI_ Z-Score was associated with a lower risk of functional impairment [OR 0.55 (0.36, 0.83) p = 0.005].

## Discussion

This is the first study to characterize age, sex and race/ethnicity-specific associations between ALMI and FMI in the NHANES population and to provide a framework through which clinical providers and investigators may adjust lean mass parameters for the confounding effects of fat. Previous studies have adjusted lean mass parameters for fat mass [8, 9]; but were limited in that they 1) did not account for significant variations in the association across age, sex, and race groups and over the range of adiposity, 2) did not capture the non-linear association between ALMI and FMI, 3) did not utilize a reference population that spans the adult age range, and 4) did not provide investigators and clinicians with the data necessary to adjust these measures without access to a control population. Thus this study represents an important advance that could lead to more widespread applications of these methods. These data now allow us to ask of a given patient: compared to individuals of similar age, sex, race, and *adiposity*, does this individual have a lower ALMI than expected?

We observed significant modification of the association between ALMI and FMI Z-Scores by age, sex, race, and adiposity. Previously described methods that do not take into account the altered association between lean and fat over the range of adiposity would be likely to over-estimate lean mass among those at the extremes of adiposity. Truly cachectic subjects with low fat mass could therefore be over-adjusted into the normal range. The current methodology takes into account the smaller association between lean and fat among those with low fat mass and therefore would result in fewer truly cachectic patients being reclassified.

The distinct presentation of results within each age, sex, and race group is vital. Previous studies have shown an altered lean-fat association by age and sex [5]. We observed that younger, female, and black subjects demonstrated greater associations between lean and fat. The biologic implications of this observation remain unclear. Elderly subjects may be less likely to demonstrate adequate muscle hypertrophy in response to weight thus explaining the declining strength of the relationship between lean and fat with age. Weight loss among the elderly might also occur preferentially within the lean compartment. Differences in sex-specific hormones related to muscle by may help to explain differences in the lean-fat association among men and women while genetic factors may play a role in the modification of the effect seen among black subjects. Environmental factors may also be implicated in these relationships. The current study design was not intended to fully characterize the pathways implicated.

There are several immediate implications of this work. Firstly, whole-body DXA estimates of muscle mass may now be adjusted for the confounding association with fat even without the availability of a control population. Secondly, a clinician who is assessing lean measures in an individual patient may now determine if lean mass deficits are out of proportion to the extent of adiposity. An obese subject who has low lean mass might be expected to be disproportionately affected compared to a thin subject with similarly low lean mass. Only adjustment for the confounding effects of fat mass will allow this distinction. Simple adjustments based on the data provided in this study can be used to easily adjust lean parameters in large datasets and thus these adjustments can be immediately applied to existing data. Estimating equations as exampled in the results can be generated from the tables provided to adjust adult body composition Z-Scores across the range of age, sex, and race. If validated, these estimating equations could be installed in software to automate these assessments and provide the information to clinicians.

This study also provides novel and comprehensive fat-adjusted definitions of “low lean mass for age” and “sarcopenia” based on distributions in a nationally representative sample. Implementation of fat-adjustment changes the categorization of many subjects who would be defined as having lean mass deficits as illustrated by the only fair to good agreement between estimates of “low lean mass for age” and “sarcopenia” before and after fat-adjustment. Reclassification from low to normal occurred primarily among younger subjects with low fat mass. It makes intuitive sense that young, thin subjects are unlikely to suffer from sarcopenia. In contrast, reclassification from normal to low occurred primarily among older subjects with greater fat mass; a group that is likely to be at high risk of functional impairment.

Previous methods of fat-adjustment have demonstrated significant improvements in correlations with mobility limitations, suggesting that adjustment for adiposity has important implications [9]. However, it is important to note that the comprehensive assessment of the associations stratified by age, sex, race, and across the range of adiposity is less likely to misclassify subjects compared to previous methods. More comprehensive assessment and validation of fat-adjusted measures in other disease states and among the elderly is a logical next step to validate the methods presented here.

An important strength of the current study is the sex- and race- specific definitions of *low lean for age* and sarcopenia. This distinction from other methods is important, since previous studies have not considered race-specific differences. The use of race-specific measures does not allow for direct comparisons across race groups in terms of the prevalence of sarcopenia. However, this comparison could potentially be accomplished by assuming all subjects are the same race and performing analyses under this assumption.

The primary limitation of this study is the lack of longitudinal measures of physical function in the NHANES participants. However, data in a cohort of adults with RA demonstrated that our new measure of ALMI_FMI_ was significantly correlated with two widely used measures of physical function, while conventional ALMI was not. Future studies are needed to further validate these measures and better understand how to utilize these fat-adjusted measures of ALM when assessing correlations with physical function and mobility limitation. Future studies might also consider ways to incorporate measures of regional adiposity in the estimation of muscle loss using a similar approach.

There are also a number of strengths of the current study. We utilized a large nationally representative population from NHANES that allowed for age-, sex-, and race-specific analyses. This study provides immediate and highly practical tools based on widely available national reference data for future investigators to be able to clarify the utility of the fat-adjusted measures presented here.

In conclusion, adjustment for the confounding association between lean and fat mass has a significant impact on the characterization and definition of lean mass deficits. Further study will be necessary to define the importance of fat-adjusted outcomes in terms of associations with functional outcomes and long-term risk.

## Supporting Information

S1 File Supplemental Tables. Table A: Characteristics of Study Samples. Table B: Mean (μ) and standard deviation (σ) of FMI by age category in the 1999–2006 NHANES sample. Table C: Mean (μ) and standard deviation (σ) of ALMI by age category in the 1999–2006 NHANES sample. Table D: Mean (μ) and standard deviation (σ) of FMI among 20–40 year old NHANES participants. Table E: Mean (μ) and standard deviation (σ) of ALMI among 20–40 year old NHANES participants.
(DOCX)
