# Supplementary material for: Development of Novel Methods to Define Deficits in Appendicular Lean Mass Relative to Fat Mass
Source: PLoS One. 2016 Oct 10;11(10):e0164385. doi: 10.1371/journal.pone.0164385 (PMC5056731; doi:10.1371/journal.pone.0164385)
Supplement: S1 File — Table A: Characteristics of Study Samples. Table B: Mean (μ) and standard deviation (σ) of FMI by age category in the 1999–2006 NHANES sample. Table C: Mean (μ) and standard deviation (σ) of ALMI by age category in the 1999–2006 NHANES sample. Table D: Mean (μ) and standard deviation (σ) of FMI among 20–40 year old NHANES participants. Table E: Mean (μ) and standard deviation (σ) of ALMI among 20–40 year old NHANES participants. (DOCX) [file pone.0164385.s001.docx]

Table A: Characteristics of Study Samples.

|  | **NHANES Cohort** | **RA Cohort** |
| --- | --- | --- |
| **N** | 14,850 | 111 |
| **Female, n (%)** | 7333 (49) | 57 (51) |
| **Racial Group** |  |  |
| **White, n (%)** | 7793 (53) | 70 (63) |
| **Black, n (%)** | 3405 (23) | 37 (33) |
| **Mexican American, n (%)** | 3652 (25) | 4 (4) |
| **Age, years^1^** | 48.9 (20-84.9) | 55.8 (19-72.7) |
| **Height, cm** | 168.2 (130.4-204.1) | 169.7 (146.1-189.8) |
| **Weight, kg** | 81.1 (25.6-218.6) | 82.6 (45.5-150.2) |
| **Body Mass Index, kg/m^2^** | 27.6 (12-76.1) | 28.8 (17.5-57.0) |
| **Fat Mass Index, kg/m^2^** | 9.4 (2.1-41.9) | 10.7 (2.4-30.1) |
| **Appendicular Lean Mass Index, kg/m^2^** | 7.7 (3.5-17.8) | 7.94 (5.1, 11.9) |
|  |  |  |

Data presented as Mean (SD) or Median (range).

Table B: Mean (μ) and standard deviation (σ) of FMI by age category in the 1999-2006 NHANES sample.

| **Males** | **20-30 (n=1311)** | | **30-40 (n=1293)** | | **40-50 (n=1436)** | | **50-60 (n=1115)** | | **60-70 (n=1264)** | | **70-90 (n=1098)** | |
| --- | --- | --- | --- | --- | --- | --- | --- | --- | --- | --- | --- | --- |
|  | μ | σ | μ | σ | μ | σ | μ | σ | μ | σ | μ | σ |
| **White** | 7.16 | 3.56 | 7.72 | 3.26 | 8.48 | 3.12 | 8.87 | 3.25 | 9.34 | 2.95 | 8.87 | 2.75 |
| **Black** | 6.95 | 4.09 | 7.74 | 3.84 | 7.62 | 3.51 | 7.97 | 3.52 | 8.50 | 3.43 | 8.29 | 2.83 |
| **MexAm** | 7.45 | 2.99 | 8.29 | 2.89 | 8.44 | 2.89 | 8.61 | 2.89 | 9.26 | 2.78 | 8.33 | 2.27 |
|  |  | |  | |  | |  | |  | |  | |
| **Females** | **20-30 (n=1195)** | | **30-40 (n=1239)** | | **40-50 (n=1435)** | | **50-60 (n=1115)** | | **60-70 (n=1305)** | | **70-90 (n=1066)** | |
|  | μ | σ | μ | σ | μ | σ | μ | σ | μ | σ | μ | σ |
| **White** | 9.86 | 4.42 | 10.83 | 4.77 | 11.60 | 4.95 | 12.37 | 4.75 | 12.92 | 4.49 | 11.82 | 3.61 |
| **Black** | 11.86 | 5.34 | 12.90 | 5.52 | 13.80 | 5.47 | 14.27 | 5.27 | 14.20 | 4.84 | 13.52 | 4.75 |
| **MexAm** | 11.23 | 4.35 | 12.08 | 4.07 | 12.91 | 4.46 | 13.62 | 4.36 | 13.15 | 3.77 | 12.45 | 4.21 |

Table C: Mean (μ) and standard deviation (σ) of ALMI by age category in the 1999-2006 NHANES sample

| **Males** | **20-30 (n=1311)** | | **30-40 (n=1293)** | | **40-50 (n=1436)** | | **50-60 (n=1115)** | | **60-70 (n=1264)** | | **70-90 (n=1098)** | |
| --- | --- | --- | --- | --- | --- | --- | --- | --- | --- | --- | --- | --- |
|  | μ | σ | μ | σ | μ | σ | μ | σ | μ | σ | μ | σ |
| **White** | 8.58 | 1.39 | 8.73 | 1.34 | 8.78 | 1.24 | 8.53 | 1.21 | 8.31 | 1.17 | 7.70 | 0.98 |
| **Black** | 9.48 | 1.74 | 9.72 | 1.67 | 9.39 | 1.50 | 9.19 | 1.52 | 8.91 | 1.41 | 8.18 | 1.21 |
| **MexAm** | 8.39 | 1.10 | 8.68 | 1.11 | 8.62 | 1.11 | 8.41 | 1.11 | 8.24 | 1.03 | 7.47 | 0.88 |
|  |  | |  | |  | |  | |  | |  | |
| **Females** | **20-30 (n=1195)** | | **30-40 (n=1239)** | | **40-50 (n=1435)** | | **50-60 (n=1115)** | | **60-70 (n=1305)** | | **70-90 (n=1066)** | |
|  | μ | σ | μ | σ | μ | σ | μ | σ | μ | σ | μ | σ |
| **White** | 6.58 | 1.14 | 6.71 | 1.25 | 6.79 | 1.35 | 6.62 | 1.24 | 6.50 | 1.20 | 6.17 | 1.02 |
| **Black** | 7.90 | 1.57 | 8.04 | 1.64 | 8.04 | 1.64 | 8.07 | 1.56 | 7.69 | 1.59 | 7.72 | 1.42 |
| **MexAm** | 6.58 | 1.21 | 6.74 | 1.15 | 6.96 | 1.25 | 6.74 | 1.26 | 6.41 | 1.10 | 6.13 | 1.04 |

* Individual Z-scores respective to sex, race and age-category for FMI and ALMI can be calculated compared to the NHANES population by using the equation:

Z= X-μ / σ

Where X represents the patient/participant FMI or ALMI of interest, and μ and σ represent the mean and standard deviation, respectively, taken from the tables above

Table D: Mean (μ) and standard deviation (σ) of FMI among 20-40 year old NHANES participants

| **Males** | **20-40 (n=2604)** | |
| --- | --- | --- |
|  | μ | σ |
| **White** | 7.46 | 3.41 |
| **Black** | 7.36 | 3.98 |
| **MexAm** | 7.83 | 2.97 |
|  |  | |
| **Females** | **20-40 (n=2434)** | |
|  | μ | σ |
| **White** | 10.40 | 4.64 |
| **Black** | 12.43 | 5.45 |
| **MexAm** | 11.66 | 4.23 |

| **Males** | **20-40 (n=2604)** | |
| --- | --- | --- |
|  | μ | σ |
| **White** | 8.66 | 1.36 |
| **Black** | 9.60 | 1.71 |
| **MexAm** | 8.52 | 1.11 |
|  |  | |
| **Females** | **20-40 (n=2434)** | |
|  | μ | σ |
| **White** | 6.65 | 1.20 |
| **Black** | 7.98 | 1.61 |
| **MexAm** | 6.66 | 1.18 |

Table E: Mean (μ) and standard deviation (σ) of ALMI among 20-40 year old NHANES participants

* Individual T-scores respective to sex and race for FMI and ALMI can be calculated compared to the NHANES population by using the equation:

T= X-μ / σ

Where X represents the patient/participant FMI or ALMI of interest, and μ and σ represent the mean and standard deviation, respectively, taken from the tables above
